# Preliminary Descriptive Study on the Conservation of Human Sweat Odor: Impact of Storage Temperature and Duration on the Stability of Volatile Organic Compounds in Sorbent Samples

**DOI:** 10.1155/ianc/7684776

**Published:** 2026-05-29

**Authors:** Michelle Leemans, Vincent Cuzuel, Laetitia Maidodou, Solène Delaplanche, Damien Steyer, Elsa Boudard, Guillaume Cognon, Etienne Audureau, Isabelle Fromantin

**Affiliations:** ^1^ Clinical Epidemiology and Ageing Unit, Institut Mondor de Recherche Biomédicale, INSERM, Paris-Est University, 94010 Créteil, France et AP-HP, Hopital Henri-Mondor, Clinical Research Unit (URC Mondor), Creteil, F-94010, France, inserm.fr; ^2^ Forensic Institute of the French Gendarmerie, Caserne Lange 5 Boulevard de l’Hautil Cedex, Cergy-Pontoise, 95001, France; ^3^ Twistaroma, Illkirch-Graffenstaden, France; ^4^ DSA, IPHC UMR7178, Université de Strasbourg, Strasbourg, France, unistra.fr; ^5^ CITHEFOR, Université de Lorraine, Nancy, EA 3452, France, univ-lorraine.fr; ^6^ Wound Care and Research Unit, Curie Institute, 26 Rue d’Ulm, Paris, 75005, France, curie.fr

**Keywords:** gas chromatography, human scent, mass spectrometry, storage, volatile organic compounds

## Abstract

Volatile organic compounds (VOCs) emitted in human matrices have gained attention for their potential in noninvasive disease detection, utilizing canine olfaction and chemical analysis. However, the stability of these VOCs under various storage conditions remains poorly understood, presenting a challenge to accurate diagnostics. This study investigates the effects of storage temperature and time on VOC conservation using three types of sorbents, Sorbstars, Twisters and Getxent, without the aim of directly comparing sorbent performance. Initially, synthetic sweat‐like mixtures were analyzed on Sorbstars and Getxent, revealing unexpected increases in signal intensity after 1 month and 2 months, which led to a shift toward human sweat samples for a more realistic assessment. Long‐term storage of human sweat samples collected on Sorbstars resulted in a marked decrease in total VOC signal intensity, with losses exceeding 80% after 18 months. Short‐term studies (2‐3 months, Sorbstar and Twisters) revealed temperature‐dependent changes in VOC signal intensity that varied by VOC and sorbent. Overall, these results highlight the importance of storage temperature and duration in VOC conservation. As a preliminary study, these findings emphasize that VOC stability should be characterized for the specific VOCs of interest and that further investigations are required to establish robust and compound‐specific conservation protocols across different storage conditions.

## 1. Introduction

Canine olfaction for detecting volatile organic compounds (VOCs) emitted by the human body has gained significant attention as a noninvasive tool for disease diagnosis, including cancer [1] and infectious diseases [2]. This method relies on the premise that specific diseases produce unique VOC profiles, which can be identified through analytical chemistry or accurately detected by trained dogs [3]. This is similar to forensic science [4], where scent matching and human identification rely on consistent odor profiles. The stability and integrity of VOC samples are thus crucial for accurate canine detection. The material used to capture the sample can influence VOC integrity, while environmental conditions like temperature, humidity, and light exposure can alter VOC profiles, ultimately affecting scent identification accuracy [5].

Research on VOC stability under various storage conditions is limited but critical. A prospective trial investigating breast cancer diagnosis by canine odorology emphasized that substantially more foundational research is required to advance the reliability and clinical applicability of canine cancer detection, particularly in the early developmental stages of this field [6]. VOC collection methods include direct sampling from biological matrices (e.g., blood, urine, and breath) and indirect sampling using absorbent materials like cotton and polymers [7]. Our systematic reviews of VOC analysis in chemical medical research [8] and canine scent detection [3] revealed considerable variability in storage practices. For chemical VOC analysis, samples have been stored at temperatures from −80°C to room temperature for durations ranging from hours to months. In canine studies, most samples are frozen, but some are stored at room temperature without a defined maximum duration. To our knowledge, only two studies have examined human VOC stability over time. One found that glass containers with minimal UVA/UVB exposure preserved VOC profiles for up to 3 weeks [5]. The other showed that frozen blood samples produced distinct VOC patterns compared to those stored at room temperature or in a refrigerator, with VOC profiles becoming more complex over time [9]. Additionally, dog performance was found to decline with aged samples compared to fresh ones [10].

Our study aimed to determine the optimal storage conditions and preservation duration of VOCs collected using polymer sorbents (Sorbstars, Getxent, and Twisters), widely used in VOC research [4, 8, 11, 12]. These sorbents were chosen because they capture a broad spectrum of VOCs from sweat, are compatible with thermal desorption–GC/MS, and minimize handling‐related artifacts (e.g., they are essentially blank prior to sampling, unlike gauze), ensuring reproducible and representative sampling. Importantly, the aim of this study was not to compare the performance of different polymers, as each polymer type may preferentially capture distinct subsets of VOCs. Our choice is further supported by systematic reviews from our team [6, 7, 12, 13], which identified polymer sorbents as practical and widely used for VOC profiling in biological matrices.

## 2. Materials and Methods

### 2.1. Subjects

Twenty healthy volunteers, aged 23–50 years, including both males and females, were recruited for this study. Participants were not subjected to specific lifestyle restrictions (e.g., diet, smoking, or cosmetic use), except for refraining from using hydroalcoholic products 24 h prior to sample collection. All samples for all tests described in this manuscript were stored in glass vials and kept in the dark, following the recommendations of Hudson et al. [5]. All participants provided consent for the collection of sweat samples only, without the collection of any personal information. Both sorbents used for sweat collection (Sorbstars and Twisters) are considered safe for contact with the skin. The protocol was reviewed internally and deemed exempt from formal ethics committee approval, given the minimal‐risk, noninvasive nature of the sampling and the fact that no personal identifying data were collected.

An overview of the different studies can be found in Table 1.

**TABLE 1:** Overview of the different conducted studies.

Study no.	Sorbent type	Sample source	Conservation temperature(s)	Analysis time points
1	Getxent, Sorbstar	Artificial sweat mixture	−18°C, 4°C, 20°C	T0, 1 week, 1 month, 2 months
2	Sorbstar	Human sweat	4°C	T0, 6 months, 12 months, 18 months
3	Sorbstar	Human sweat	4°C, 20°C	T0, 1 week, 1 month, 2 months, 3 months
4	Twister	Human sweat	4°C, 20°C	T0, 1 week, 1 month, 2 months

### 2.2. Artificial Mixture and Sample Preparation

All sorbent devices were handled using sterilized forceps; personnel, but not the sampled person wore, gloves and lab coats during sample collection; samples were securely stored, and all materials were disposed of following biohazard safety protocols.

*Study 1 | Artificial mixture (Sorbstars and Getxent)*: An artificial mixture composed of 62 compounds, selected for their presence in human sweat and association with various diseases, was utilized in this study (Supporting Table 1). Each Sorbstar (2 cm‐long, 2‐mm‐diameter polymeric, Action Europe, France) was spiked with 1 μL of a 100 ppm solution of this mixture and 5 μL of a 100 ppm mixture on Getxent tubes (Biodesiv, Switzerland). Four technical replicates and a blank were analyzed at each time point: initial (T0), 1 week, 1 month, and 2 months. Samples were stored at −20°C, 4°C, or +20°C.

### 2.3. Sweat Sampling Protocol and Experimental Setup

*Study 2 | Long-Term Human Sweat Experiment (Sorbstars, up to 18 months)*: Sweat collection was performed using Sorbstars. Ten participants (biological replicates) were instructed to rub 12 Sorbstars in their palms for 30 min without preconditioning or handwashing. A long time period was chosen to ensure collection of a maximum amount of VOCs. Following collection, Sorbstars were stored in 2 mL amber glass vials with PTFE/white silicone septa and kept at 4°C. Samples were analyzed at four time points: initial (T0), 6 months (T1), 12 months (T2), and 18 months (T3). Each sample was analyzed in technical triplicate at each time point, with a blank prepared for internal control.

*Study 3 | Short-Term Human Sweat Experiment (Sorbstars, up to 3 months)*: In this experiment, five participants (biological replicates) rubbed 12 Sorbstars in their palms for 30 min without preconditioning or hand washing. After collection, Sorbstars were stored in 2 mL amber glass vials with PTFE/white silicone septa, at either 4°C or 20°C, and analyzed at four time points: initial (T0), 7 days (T1), 1 month (T2), 2 months (T3), and 3 months (T4). Each sample was analyzed in technical triplicates at each time point, with a blank included for internal control.

*Study 4 | Short-Term Human Sweat Experiment (Twisters, up to 2 months)*: Sweat was collected using 1 cm magnetic stir bars (Twisters, Gerstel, Germany) coated with 1 mm of Polydimethylsiloxane (PDMS). Prior to use, Twisters were preconditioned by nitrogen flow (99.999%) at 250°C for 1 h (TC‐2, Gerstel). Five participants (biological replicates) washed their hands with neutral soap (Le Petit Marseillais) 1 h before sampling. Each participant held 12 Twisters in their palms for 1 h. After collection, Twisters were stored in 2 mL amber glass vials with PTFE/white silicone septa at 4°C or 20°C and analyzed at four time points: initial (T0), 2 weeks (T1), 1 month (T2), and 2 months (T3). Samples were analyzed in technical triplicates, with blanks prepared for internal control.

### 2.4. Analytical Devices

*Study 1 | Getxent (Artificial Mixture)*: After sample collection, Getxent tubes were thermodesorbed using a TD100‐xr thermodesorption system (Markes International Ltd, UK). Volatile compounds were purged from the sorbents using the carrier gas, and the components were concentrated by adsorption on a built‐in general‐purpose graphitized carbon trap at −30°C. After incubation at 100°C and a 20 mL/min purge for 20 min, the analytes were desorbed and transferred onto a transfer line heated to 200°C, followed by chromatographic separation on a DB‐1MS column (20 m × 0.18 mm, 0.72 μm) in a GC‐MS Q2010Plus (Shimadzu, France). The initial temperature was set at 40°C for 4 min, ramped to 300°C at 20°C/min, and held at 300°C for 6 min. The MS operated in scan mode (29–250 m/z, 70 eV ionization).

*Study 1 | Sorbstars (Artificial Mixture)*: Sorbstar samples were thermodesorbed using the same TD100‐xr system. Volatile components were purged at 250°C and concentrated on a graphitized carbon trap at −30°C. After incubation at 250°C and a 20 mL/min purge for 20 min, the concentrated substances were transferred to a GC‐MS Q2010Plus (Shimadzu, France) for analysis. Chromatographic separation was achieved using a DB‐1MS column (20 m × 0.18 mm, 0.72 μm). The GC method followed the same temperature program as the Getxent tubes. The MS operated in scan mode (29–250 m/z, 70 eV ionization).

*Study 2 | Long-Term Human Sweat Experiment (Sorbstars, up to 18 months)*: For long‐term analysis, Sorbstars were thermodesorbed using a VSP4000 purge‐and‐trap system (Innovative Messtechnik GmbH, Germany). Volatile compounds were purged from the Sorbstars with carrier gas, and analytes were adsorbed on a Tenax TA trap at −30°C. After incubation at 190°C and a 20 mL/min purge for 20 min, the compounds were transferred onto a DB‐1MS GC column (30 m × 0.25 mm, 0.25 μm) and a DB‐1701 column (1.5 m × 0.1 mm, 0.1 μm) in a GC × GC‐MS Q2010Plus (Shimadzu, France). The initial temperature was set at 40°C for 1 min, ramped to 250°C at 2.5°C/min, and held at 250°C for 1 min. The MS was operated in scan mode (29–250 m/z, 70 eV ionization), and the sampling frequency was 50 Hz. The modulation was performed with an N2‐cooled Zoex ZX1 thermal modulator, and the modulation time was set at 8 s.

*Study 3 | Short-Term Human Sweat Experiment (Sorbstars, up to 3 months)*: Thermodesorption was performed using the VSP4000 purge‐and‐trap system (Innovative Messtechnik GmbH, Germany) and analyzed using a GC‐MS Q2010Plus (Shimadzu, France) with a DB‐1MS column (30 m × 0.25 mm, 0.25 μm). The GC program followed the conditions as described for the long‐term experiment, with MS operated in scan mode (29–250 m/z, 70 eV ionization).

*Study 4 | Short-Term Human Sweat Experiment (Twisters, up to 2 months)*: Samples were analyzed by direct thermal desorption using a GC‐ToF‐MS system (Agilent 7890B GC, Leco Pegasus BT‐ToF MS). Chromatographic separation was carried out on a polar capillary column (DB‐WAX, Agilent Technologies) (30 m × 0.25 mm x 0.25 μm). The GC oven temperature program started at 40°C (held for 2 min), followed by a temperature ramp of 5°C/min up to 250°C (held for 5 min). The MS operated in scan mode (30–400 m/z) with an oven temperature program: 40°C (2 min), ramped at 5°C/min to 250°C (6 min). Desorption was conducted in two steps: primary desorption in a Thermal Desorption Unit (TDU, Gerstel) and secondary desorption in a Cooled Injector System (CIS, Gerstel) equipped with a Tenax TA liner. The TDU initial temperature was set at 40°C, then increased to 250°C at 120°C/s for 8 min, while the CIS initial temperature was set at −10°C and ramped to 300°C at 12°C/s for 5 min.

### 2.5. Data Processing

*Studies 1 and 3| Getxent (Artificial Mixture) and Sorbstars (Artificial Mixture and Short-Term Human Sweat Experiment)*: Raw data were processed using GC‐MS Solution (Shimadzu) software. For the short‐term human sweat experiment, compounds were selected based on their frequent detection across samples, and peaks with a signal/noise ratio > 10 were considered detected, and VOCs were identified using the NIST17 database, with a match factor ≥ 80%.

*Study 2 | Long-Term Human Sweat Experiment (Sorbstars)*: Chromatogram processing followed the method of Cuzuel et al., 2018 [14]. Briefly, a preliminary manual analysis of 25 chromatograms led to a customized library of 761 compounds with mass spectra. Compound identification was based on mass spectra and retention times, using the NIST 14 library for reference (≥ 80% similarity) when possible. Each chromatogram was represented by these 761 compounds and their peak intensities, normalized to account for total signal variation.

*Study 4 | Short-Term Human Sweat Experiment (Twisters)*: Raw data were processed using MassHunter Quantitative Analysis (Agilent). To reduce contamination, blank desorption tubes were injected before and after sample analysis. Quantifier and Qualifier ion ratios were confirmed within a ± 20% tolerance to validate compound identification. VOCs detected in blanks (sampling room air) were excluded from analysis. For Twisters, the eight most frequently detected compounds and peaks with a signal/noise ratio > 10 were considered detected, and VOCs were identified using the NIST17 database, with a match factor ≥ 80%.

### 2.6. Statistical Analysis

For all studies, the effect of time on VOC measurements was evaluated using linear mixed‐effects models to account for repeated measurements within individuals.

• For Study 2 (long‐term sweat, up to 18 months), the dependent variable was the total ion current (TIC), conservation time was included as a fixed effect, and individual was included as a random intercept.
• For Studies 3 and 4 (short‐term sweat, Sorbstars, and Twisters), the dependent variable was the sum of selected VOC peak intensities per individual at each time point, with time as a fixed effect and individual as a random intercept.

All models were fitted using restricted maximum likelihood. Model assumptions were checked visually using residual diagnostics. Statistical significance was assessed at *α* = 0.05.

For visualization, variability was represented using the standard error of the mean (SEM): for each individual and time point, SEM was calculated as the standard deviation of technical replicate measurements divided by the square root of the number of technical replicates. Shaded areas in figures represent ±SEM around the mean.

## 3. Results

### 3.1. Artificial Sweat‐Like Mixture

Measurements taken after 1 month revealed an increase in signal intensity for all compounds in the mixture on both sorbents, across all tested storage conditions (Supporting Figure 1). This increase in signal intensity persisted through the 2 month time point. However, due to unforeseen technical difficulties, further measurements could not be carried out. In response to these results, we revised the experimental design to more accurately address the original research question regarding the influence of storage temperature and conservation time. Shifting the focus from the synthetic mixture, the study was adapted to use human sweat, providing a more realistic scenario for further investigation.

### 3.2. Long‐Term Conservation of Hand Sweat VOCs on Sorbstars

#### 3.2.1. Descriptive Analysis

**FIGURE 1:**
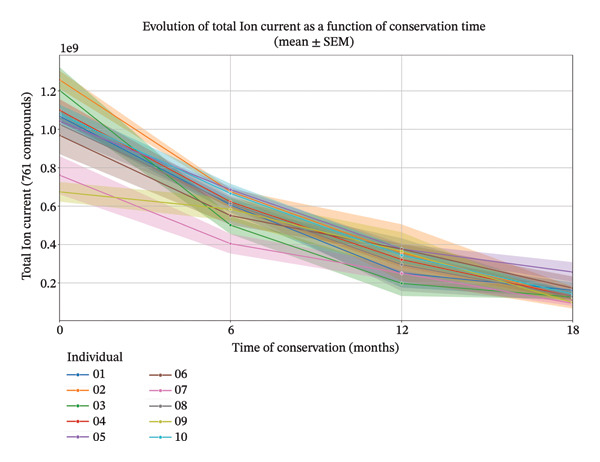
Study 1: Total ion current evolution over conservation time (0, 6, 12, 18 months) for 10 different individuals with SEM for each individual. Shaded areas represent the SEM, showing variability across technical replicates.

Mean TIC values decreased progressively with conservation time. Average TIC declined from approximately 1.02 × 10^9^ at the first time point to 1.43 × 10^8^ at the final time point, corresponding to a cumulative reduction of more than 85%. This decrease was observed consistently across individuals, although absolute TIC levels varied between individuals (Figure 1).

#### 3.2.2. Linear Mixed‐Effects Model

The linear mixed‐effects model included 119 observations from 10 individuals and successfully converged. Conservation time had a strong and statistically significant negative effect on TIC (*β* = −2.90 × 10^8^ per time point, SE = 1.13 × 10^7^, *z* = −25.7, *p* < 0.001). The 95% confidence interval for this effect did not include zero (−3.12 × 10^8^ to −2.68 × 10^8^), indicating a robust decline in TIC with increasing conservation time.

The random intercept variance was substantial (Group Var = 3.23 × 10^15^), indicating pronounced differences in baseline TIC levels between individuals. Despite this interindividual variability, the direction and magnitude of the temporal decrease were consistent across individuals.

### 3.3. Short‐Term Conservation of Hand Sweat VOCs at Different Temperatures

#### 3.3.1. Sorbstars

Our analysis focused on the six most abundant compounds identified across individuals (Figures 2, 3(a), and 3(b)). For two out of the five individuals, little compounds were detected. However, for the other three individuals, different components were identified within the sweat samples. Interestingly, different signal intensity profiles were observed for similar components from the same individuals stored at different temperatures.

FIGURE 2Study 3: VOC behavior over a 90 day period for five different individuals using Sorbstar as the capturing material at 20°C (a) and at 4°C (b). The graph shows the temporal variation in VOC signal intensity, with mean values represented by solid lines and the standard error of the mean (SEM) displayed as shaded areas around each line. Different compounds are color‐coded, and the legend corresponds to each compound.

**Figure:**
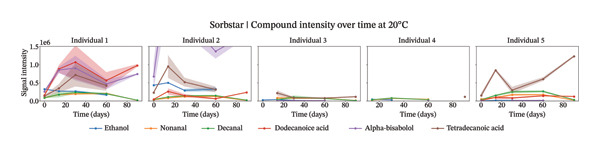
(a)

**Figure:**
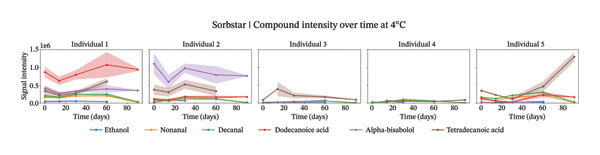
(b)

FIGURE 3Study 3: VOC behavior over a 90‐day period for five different individuals using Sorbstar as the capturing material at 20°C (a) and at 4°C (b) over a 60‐day period for five different individuals. The graphics show the temporal variation in the sum of selected VOC signal intensities of selected compounds. For Sorbstar, the following compounds were summed: ethanol, nonanal, decanal, dodecanoic acid, alpha‐bisabolol, and tetradecanoic acid.

**Figure:**
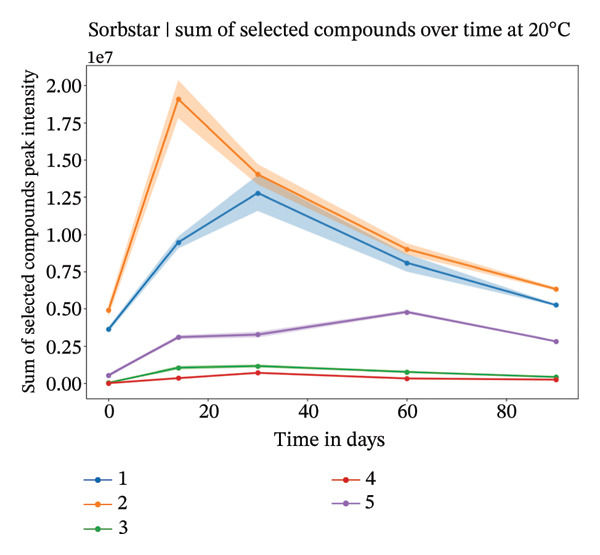
(a)

**Figure:**
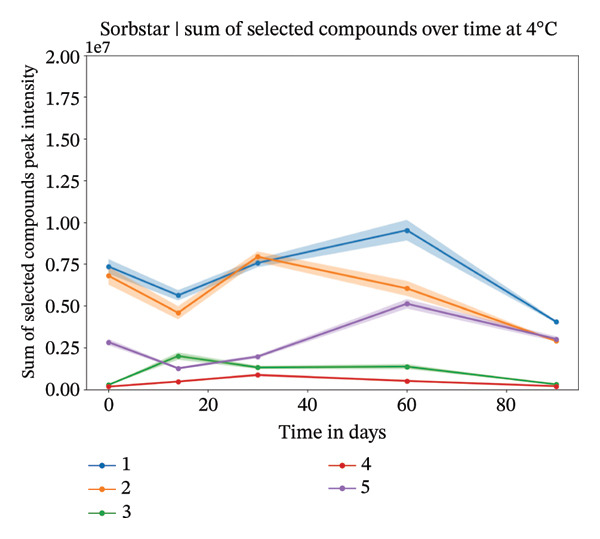
(b)

At 4°C, the sum of the selected compounds per individual remained relatively stable over time. This was confirmed by a linear mixed‐effects model, which showed a nonsignificant effect of time on the total peak intensity (*β* = −6,850, *p* = 0.457), indicating minimal degradation of VOCs under cold storage.

At 20°C, while the signal intensities were slightly higher for some individuals, the linear mixed‐effects model again showed no significant change over the short‐term period studied (*β* = −7,385, *p* = 0.710). The *β* coefficients suggest a slightly faster decline at 20°C than at 4°C, but neither was statistically significant. These results suggest that, although individual compound intensities may fluctuate, the overall abundance of the main VOCs is largely maintained within the 3‐month period. The VOC peak intensity behavior for each compound across individuals is presented in Supporting Figure 2.

#### 3.3.2. Twisters

Our analysis focused on the eight most abundant compounds identified across individuals (Figures 4(a) and 4(b)). For one out of the five individuals, a small amount of compounds were detected. However, for the other four individuals, more components were identified within the sweat samples. VOC degradation was observed during the first 2 weeks, after which it stabilized (Figures 5(a) and 5(b)). This observation was supported statistically by linear mixed‐effects models applied to the sum of the selected compounds per individual over time. Based on the *β* coefficients from linear mixed‐effects models, VOCs declined potentially slightly faster at 4°C than at 20°C (4°C: *β* = −22,214,194, *p* value = 0.006; 20°C: *β* = −18,324,002, *p* value = 0.007), though degradation rates varied between individuals. High between‐individual variability suggests that some individuals’ samples may degrade faster or slower than others. The VOC peak intensity behavior for each of the compounds across the different individuals can be found in Supporting Figure 3.

FIGURE 4Study 4: VOC behavior over a 60 day period for five different individuals using Twister as the capturing material at 20°C (a) and at 4°C (b). The graph shows the temporal variation in VOC signal intensity, with mean values represented by solid lines and the standard error of the mean (SEM) displayed as shaded areas around each line. Different compounds are color‐coded, and the legend corresponds to each compound.

**Figure:**
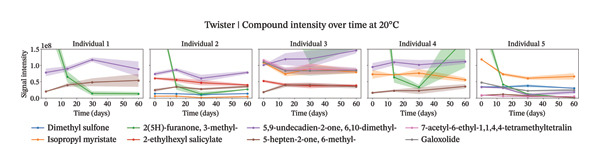
(a)

**Figure:**
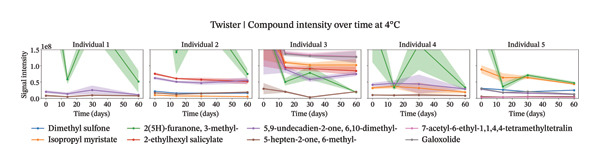
(b)

FIGURE 5Study 4: VOC behavior over a 90‐day period for five different individuals using Twister as the capturing material at 20°C (a) and at 4°C (b). The graphics show the temporal variation in the sum of selected VOC signal intensities of selected compounds. For Twister, the following compounds were summed: isopropyl myristate; 2(5H)‐furanone, 3‐methyl; 2‐ethylhexyl salicylate; 5,9‐undecadien‐2‐one,6,10‐dimethyl ; 5‐hepten‐2‐one, 6‐methyl‐; 7‐acetyl‐6‐ethyl‐1,1,4,4‐.

**Figure:**
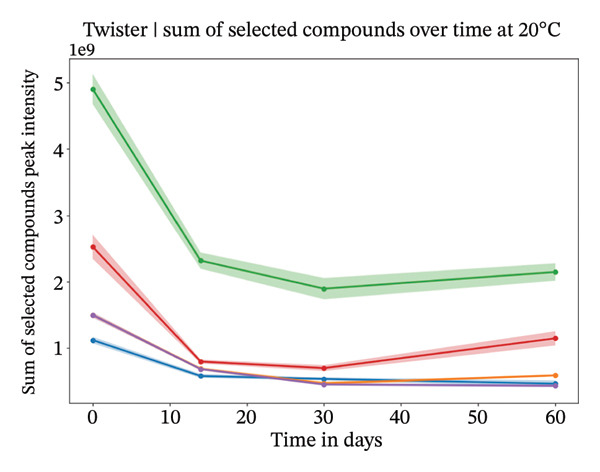
(a)

**Figure:**
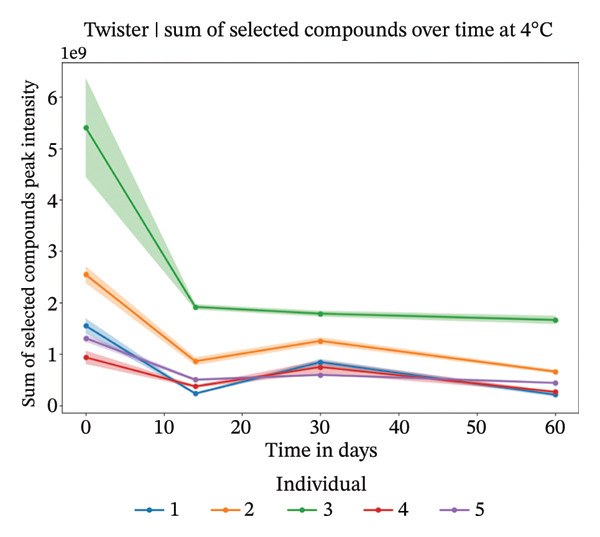
(b)

## 4. Discussion

### 4.1. Artificial Sweat‐Like Mixture

Initially, an artificial sweat‐like mixture (Study 1) was used because the composition and concentration of compounds were well‐defined, allowing for better control and potentially clearer observation of conservation patterns over time. Unexpectedly, a substantial increase in signal intensity was observed after 1 month of storage, contradicting the anticipated trend of progressive VOC degradation. Despite considering several plausible explanations, including sorption‐desorption processes and potential interaction between VOCs and the sorbent material, no definitive mechanistic explanation could be established based on the available data. A more rigorous mechanistic investigation would have required additional targeted experiments that were beyond the scope of the present study. Additionally, the higher short‐term signals in synthetic mixtures likely reflect the absence of the complex biological matrix found in real sweat, which contains proteins, salts, lipids, and microbial by‐products [13]. Another hypothesis is that without the complex biological matrix, VOCs potentially behave unrealistically, increasing in signal rather than following the degradation trends expected in real samples—highlighting why we shifted focus toward human sweat for biologically relevant observations.

Importantly, this increase in signal intensity was consistently reproduced in two independent experiments using three different GC‐MS instruments, indicating that the observation was not attributable to analytical or instrumental error. Throughout this work, the investigation of VOC storage revealed multiple methodological challenges, highlighting the complexity of an issue initially assumed to be straightforward. By transparently reporting these observations and limitations, we aim to contribute to the establishment of practical guidelines for VOC‐related research in both analytical chemistry and canine detection fields. While our findings provide initial insights, further dedicated studies are required to validate these results and to elucidate the underlying mechanisms.

### 4.2. Long‐Term Conservation of Hand Sweat VOCs

Sweat sampling from 10 individuals revealed a pronounced and consistent decrease of VOC‐related signal intensity over the storage period. Total ion current, used as a proxy for global VOC abundance, declined markedly with increasing conservation time, with a substantial loss already evident after 6 months of storage (Study 2). This progressive reduction continued across subsequent time points, demonstrating that human VOCs are not stable over extended periods, even when samples are stored under controlled conditions.

These findings are consistent with previous observations reported by Hudson et al. [5] and underscore the challenges associated with long‐term VOC conservation, including storage at 4°C in the dark and in sealed containers. While such a degradation may not critically affect diagnostic applications, which typically require rapid analysis following sampling, it is relevant even for diagnostics because canines are trained on stored training samples, some of which may be kept for extended periods or reused. The potential impact of repeated opening and closing of storage jars on VOC degradation represents an additional topic that requires investigation. In forensic contexts, where extended preservation is essential, the observed temporal instability of VOC profiles challenges the feasibility of maintaining a reliable “human odor library” using Sorbstars. Whether these findings can be extrapolated to other sorbent types remains unknown, highlighting the need for further studies to evaluate long‐term VOC stability across different sorbents. The pronounced between‐individual variability further emphasizes that VOC degradation rates differ between donors, adding complexity to strategies for long‐term preservation.

### 4.3. Short‐Term Conservation of Hand Sweat VOCs at Different Temperatures

Individual variability was notable, with some participants showing higher VOC levels and/or more VOCs than others. This variability further highlights the complexity of VOC conservation, as different sorbents, temperatures, individuals, and compounds may all behave differently. It is important to note that this analysis was based on only five individuals and a limited number of compounds, meaning these findings should be interpreted with caution.

In this study (Studies 3 and 4), short‐term VOC conservation from human sweat was evaluated over 2‐3 months at two storage temperatures (4°C and 20°C) using two types of sorbents: Sorbstars and Twisters. Although Sorbstars and Twisters are based on the same stationary phase, they are proprietary materials, and detailed information regarding their material composition, surface characteristics, and internal structure is not publicly available. Consequently, while differences in VOC behavior were observed, attributing these patterns to specific physicochemical properties would be speculative, and this study therefore focuses on comparative practical performance rather than mechanistic deconvolution.

For Sorbstars, VOCs appeared relatively stable during this period; however, some differences in VOC patterns emerged with higher storage temperatures potentially inducing microbial activity or other temperature‐dependent changes [15, 16]. Notably, at 20°C, most compounds showed an initial increase in signal intensity during the first 1‐2 months, followed by a subsequent decrease. This suggests that temperature may influence VOC conservation, likely through microbial or chemical processes that are more active at room temperature [17, 18]. Despite these fluctuations for individual compounds, the total abundance of the main VOCs for Sorbstars remained largely stable over time at both temperatures, as confirmed by linear mixed‐effects modeling. These studies underscore the complexity of VOC stability across different storage temperatures, aligning with findings from Forbes et al. [9].

Comparing these short‐term results to long‐term conservation for Sorbstar, it is possible that VOC degradation accelerates over extended storage, potentially following a time‐dependent, nonlinear process. Cumulative effects such as volatilization, chemical breakdown, adsorption to container materials, or slow matrix‐induced reactions may contribute to the sharper decline in signal intensity observed over longer durations. This remains a hypothesis that should be tested in future studies. In addition, certain chemical classes (e.g., aldehydes and fatty acids) are known to be more chemically reactive and therefore potentially more susceptible to degradation processes such as oxidation or hydrolysis than more stable compounds [19, 20]. Although these class‐dependent effects were not specifically investigated here, they may partly explain the differential decay rates observed across VOCs and represent an important hypothesis to be addressed in future targeted studies.

For the short‐term Twisters experiment, VOC degradation occurred within the first 2 weeks, after which it stabilized. For linear mixed‐effects models applied to the sum of compound intensities per individual confirmed that degradation occurred. Pronounced between‐individual variability further suggests that some samples degrade more rapidly than others, likely due to differences in initial VOC composition or matrix effects.

Larger sample sizes and a broader range of compounds will be required to confirm these trends and to better understand the interactions between sorbent type, temperature, and individual variability. Additionally, different compounds may have varying degrees of stability depending on their chemical nature and how they interact with the sorbent, suggesting that sorbent selection and storage conditions may need to be tailored depending on the VOCs of interest.

Furthermore, sweat composition varies across body zones due to differences in gland activity, skin microbiota, and local blood flow [21], leading to distinct electrolyte and metabolite profiles in sweat from areas like the forehead, back, and palms. This highlights the need to optimize analysis based on the collection site and the specific VOCs involved, which may require different sorbents or sampling methods for improved detection. Additionally, in the context of disease detection, it remains unclear whether a single VOC or a mixture of VOCs serves as the key biomarker. Therefore, it is essential to maintain a broad detection strategy by potentially combining different sorbents that capture various VOCs with differing affinities and release profiles. Similar challenges would apply when analyzing other matrices, such as saliva, urine, and interstitial fluid, which reflect localized metabolic states relevant to various diseases or other applications.

The potential impact of different storage conditions on canine detection accuracy was not addressed in this study. However, it is important to note that Schoon [10] observed reduced canine performance when working with older samples.

Prospective canine odorology studies in cancer detection highlight that the field is still in an early developmental stage and requires substantially more foundational and methodological research [6]. This preliminary study provides a structured comparison of VOC conservation across different sorbents, storage conditions, and sample types, combining controlled artificial mixtures with real human sweat. It also highlights the complexity of the subject: when investigating specific VOCs, it is critical to determine which sorbent best captures the compounds of interest, the optimal storage temperature, and the maximum duration for which samples can be preserved. While this requires considerable effort, such systematic evaluation is essential in contexts like cancer research, where VOC concentrations are extremely low and mixtures are highly complex. Limitations of the study include a small sample size and a restricted number of VOCs, limiting generalizability. Unexpected results with artificial mixtures suggest possible uncontrolled sorbent‐related processes. Additionally, factors such as sampling site variability and the impact on canine detection were not explored and should be addressed in future research.

## 5. Conclusion

This preliminary descriptive study provides insights into the factors influencing VOC conservation of human sweat, highlighting the complex interplay between sorbent type, storage temperature, and individual variability. VOC signal intensity showed substantial degradation after 6 months, suggesting that samples should be analyzed within this period to ensure reliable results. Over shorter periods (2‐3 months), VOCs remained relatively stable, though temperature‐dependent changes were noted. Larger studies are needed to refine guidelines for VOC analysis. Given the complexity of VOC conservation, it would be beneficial for each study to conduct a preliminary investigation on the optimal conservation methods for the VOCs of interest to improve reliability and reproducibility, a need that is echoed in canine odorology research on cancer detection, which points to the importance of further foundational and methodological work in this emerging field.

## Author Contributions

Conceptualization: Michelle Leemans, Vincent Cuzuel; methodology: Michelle Leemans, Vincent Cuzuel; data collection: Laetitia Maidodou, Solène Delaplanche, Elsa Boudard, Michelle Leemans, Vincent Cuzuel; formal analysis: Laetitia Maidodou, Solène Delaplanche, Michelle Leemans, Vincent Cuzuel; investigation: Michelle Leemans, Vincent Cuzuel; data curation: Laetitia Maidodou, Solène Delaplanche, Michelle Leemans, Vincent Cuzuel; writing–original draft: Michelle Leemans, Vincent Cuzuel; writing–review and editing: Michelle Leemans, Vincent Cuzuel, Isabelle Fromantin, Guillaume Cognon, Etienne Audureau; Visualization: Michelle Leemans; supervision: Vincent Cuzuel, Michelle Leemans; funding acquisition: Isabelle Fromantin; project administration: Michelle Leemans.

## Funding

This study was funded by the Royal Canin Foundation and a sponsorship from Seris Security.

## Disclosure

The funders had no role in the study design, data collection, analysis, or interpretation of the results.

## Conflicts of Interest

The authors declare no conflicts of interest.

## Supporting Information

Additional supporting information can be found online in the Supporting Information section.

## Supporting information


**Supporting Information** Supporting Table 1: Composition of artificial mixture. Supporting Table 2: Retention times (min) and characteristics ions (m/z) selected for the VOCs identified in the samples; Quantifier refers to the ion used for the relative quantification, and Qualifier refers to the ion selected to confirm the VOC identification. Supporting Figure 1 | Study 1: VOC behavior over a 60‐day period for the artificial sweat mixture using Getxent (A) and Sorbstar (B) as the capturing material at ‐20°C, 4°C and at 20°C. The graph shows the temporal variation in VOC signal intensity, with mean values represented by solid lines and the standard error of the mean (SEM) displayed as shaded areas around each line. Different compounds are color‐coded, and the legend corresponds to each compound. Supporting Figure 2 | Study 3: VOC behavior over a 90‐day period for different compounds using Sorbstar as the capturing material (a) at 20°C and (b) at 4°C. Each graph displays the temporal variation in VOC signal intensity for individual compounds, with separate lines representing different individuals. The mean values are depicted by solid lines, while the standard error of the mean (SEM) is shown as shaded areas around each line. Different individuals are color‐coded, and the legend corresponds to each individual. Supporting Figure 3 | Study 4: VOC behavior over a 60‐day period for different compounds using Twister as the capturing material (a) at 20°C and (b) at 4°C. Each graph shows the temporal variation in VOC signal intensity for individual compounds, with separate lines representing different compounds. The mean values are depicted by solid lines, and the standard error of the mean (SEM) is shown as shaded areas around each line. Different individuals are color‐coded, and the legend corresponds to each individual.

## Data Availability

The datasets are publicly available on Figshare: Leemans, Michelle (2026). Data Study 1 | Artificial mixture using Getxent and Sorbstar. figshare. Dataset. https://doi.org/10.6084/m9.figshare.31268302.v1. Leemans, Michelle (2026). Data study 2 | Long term sorbstar. figshare. Dataset. https://doi.org/10.6084/m9.figshare.31276990.v1. Leemans, Michelle (2026). Data study 3 | Short term Sorbstar. figshare. Dataset. https://doi.org/10.6084/m9.figshare.31268557.v1. Leemans, Michelle (2026). Data Study 4 | Twisters short‐term. figshare. Dataset. https://doi.org/10.6084/m9.figshare.31267885.v1.
