# Supplementary material for: Preliminary Descriptive Study on the Conservation of Human Sweat Odor: Impact of Storage Temperature and Duration on the Stability of Volatile Organic Compounds in Sorbent Samples
Source: Int J Anal Chem. 2026 May 29;2026:7684776. doi: 10.1155/ianc/7684776 (PMC13239232; doi:10.1155/ianc/7684776)
Supplement: Supplementary file 1 — Supporting Information Supporting Table 1: Composition of artificial mixture. Supporting Table 2: Retention times (min) and characteristics ions (m/z) selected for the VOCs identified in the samples; Quantifier refers to the ion used for the relative quantification, and Qualifier refers to the ion selected to confirm the VOC identification. Supporting Figure 1 | Study 1: VOC behavior over a 60‐day period for the artificial sweat mixture using Getxent (A) and Sorbstar (B) as the capturing material at ‐20°C, 4°C and at 20°C. The graph shows the temporal variation in VOC signal intensity, with mean values represented by solid lines and the standard error of the mean (SEM) displayed as shaded areas around each line. Different compounds are color‐coded, and the legend corresponds to each compound. Supporting Figure 2 | Study 3: VOC behavior over a 90‐day period for different compounds using Sorbstar as the capturing material (a) at 20°C and (b) at 4°C. Each graph displays the temporal variation in VOC signal intensity for individual compounds, with separate lines representing different individuals. The mean values are depicted by solid lines, while the standard error of the mean (SEM) is shown as shaded areas around each line. Different individuals are color‐coded, and the legend corresponds to each individual. Supporting Figure 3 | Study 4: VOC behavior over a 60‐day period for different compounds using Twister as the capturing material (a) at 20°C and (b) at 4°C. Each graph shows the temporal variation in VOC signal intensity for individual compounds, with separate lines representing different compounds. The mean values are depicted by solid lines, and the standard error of the mean (SEM) is shown as shaded areas around each line. Different individuals are color‐coded, and the legend corresponds to each individual. [file IANC-2026-7684776-s001.docx]

# Supplementary Tables

**Supplementary Table 1:** Composition of artificial mixture.

**Supplementary Table 2:** Retention times (min) and characteristics ions (m/z) selected for the VOCs identified in the samples; Quantifier refers to the ion used for the relative quantification, and Qualifier refers to the ion selected to confirm the VOC identification

# Supplementary Figures


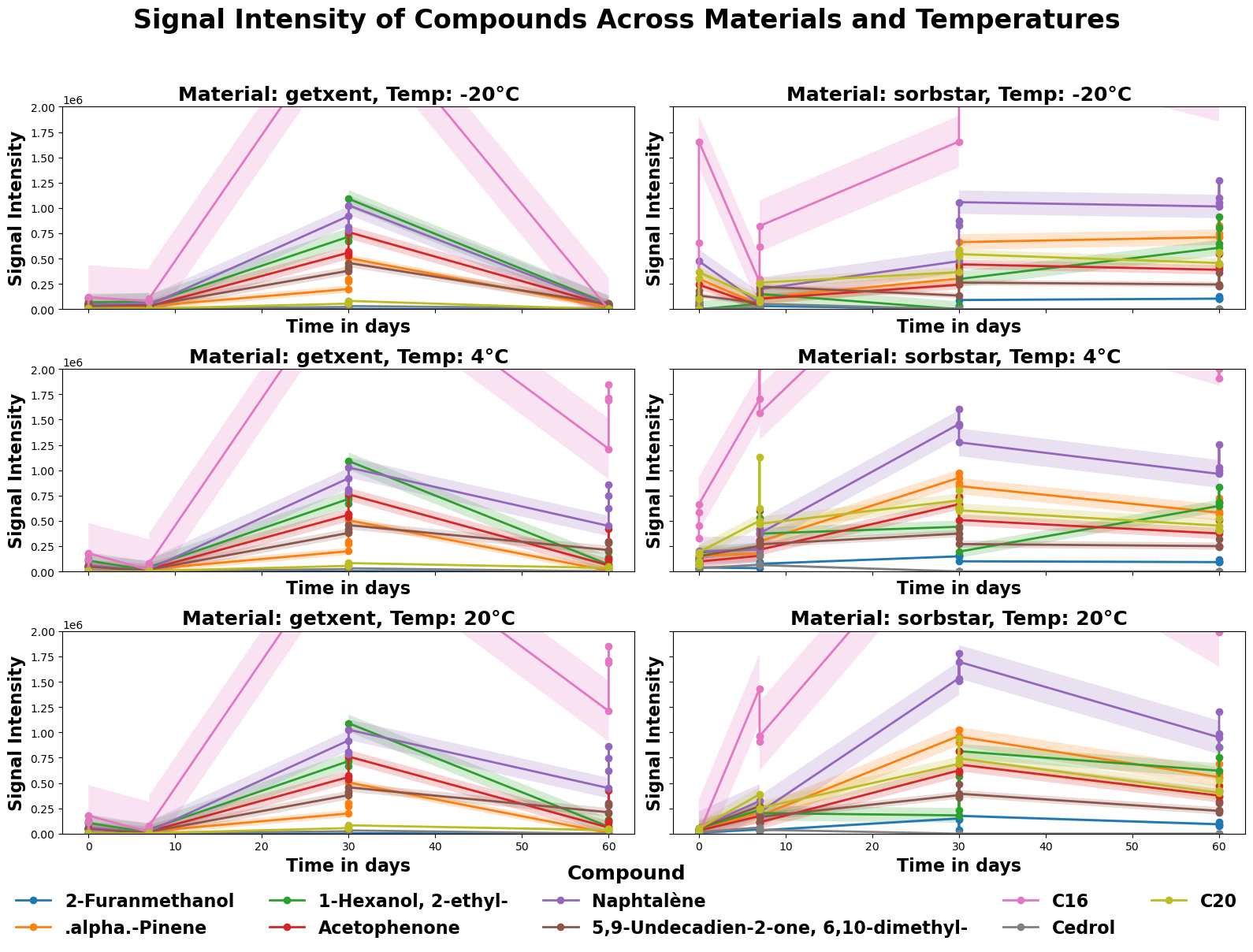


**Supplementary Figure 1 | Study 1:** VOC behavior over a 60-day period for the artificial sweat mixture using Getxent (A) and Sorbstar (B) as the capturing material at -20°C, 4°C and at 20°C. The graph shows the temporal variation in VOC signal intensity, with mean values represented by solid lines and the standard error of the mean (SEM) displayed as shaded areas around each line. Different compounds are color-coded, and the legend corresponds to each compound.


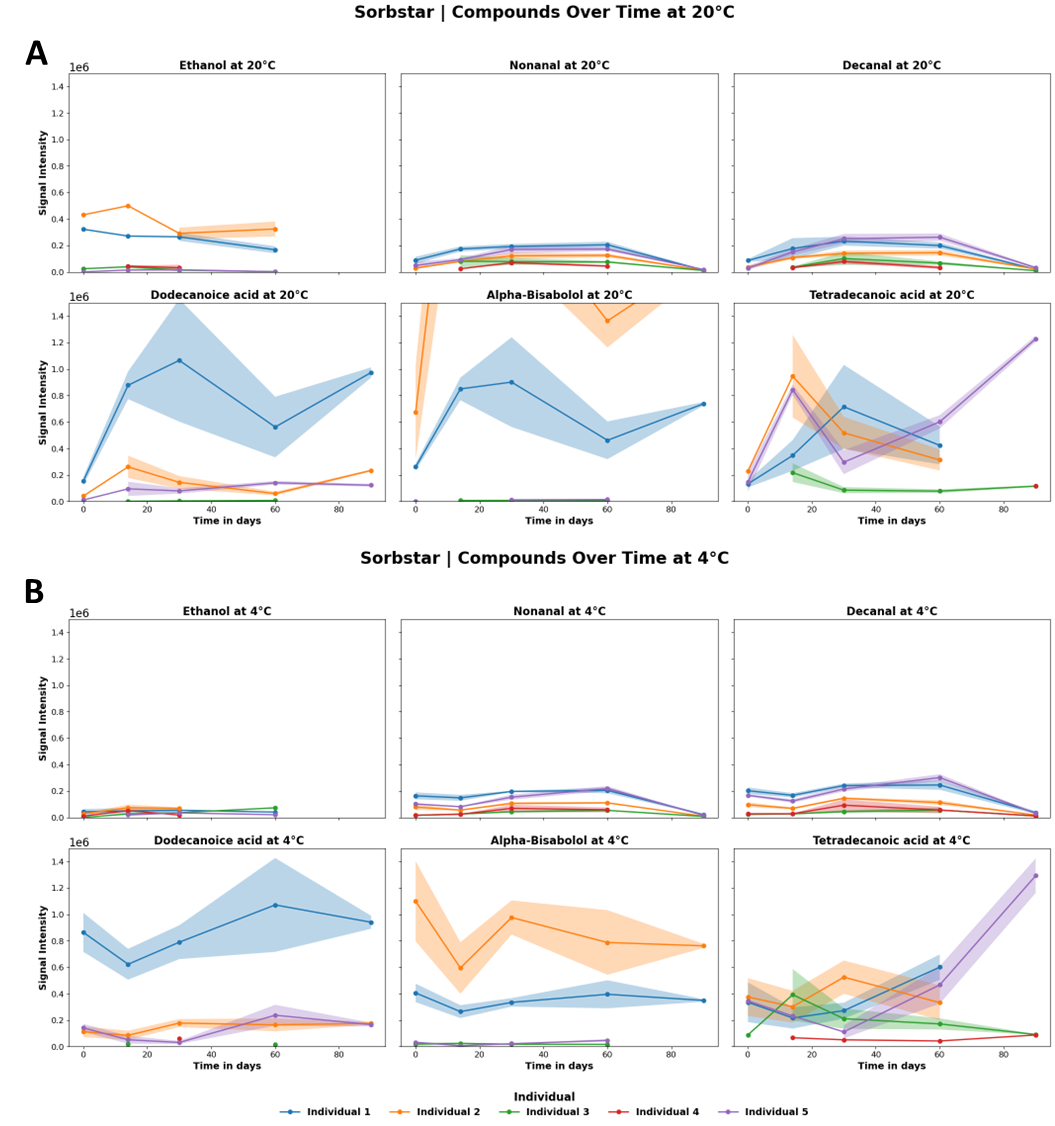
**Supplementary Figure 2 | Study 3:** VOC behavior over a 90-day period for different compounds using Sorbstar as the capturing material (a) at 20°C and (b) at 4°C. Each graph displays the temporal variation in VOC signal intensity for individual compounds, with separate lines representing different individuals. The mean values are depicted by solid lines, while the standard error of the mean (SEM) is shown as shaded areas around each line. Different individuals are color-coded, and the legend corresponds to each individual.


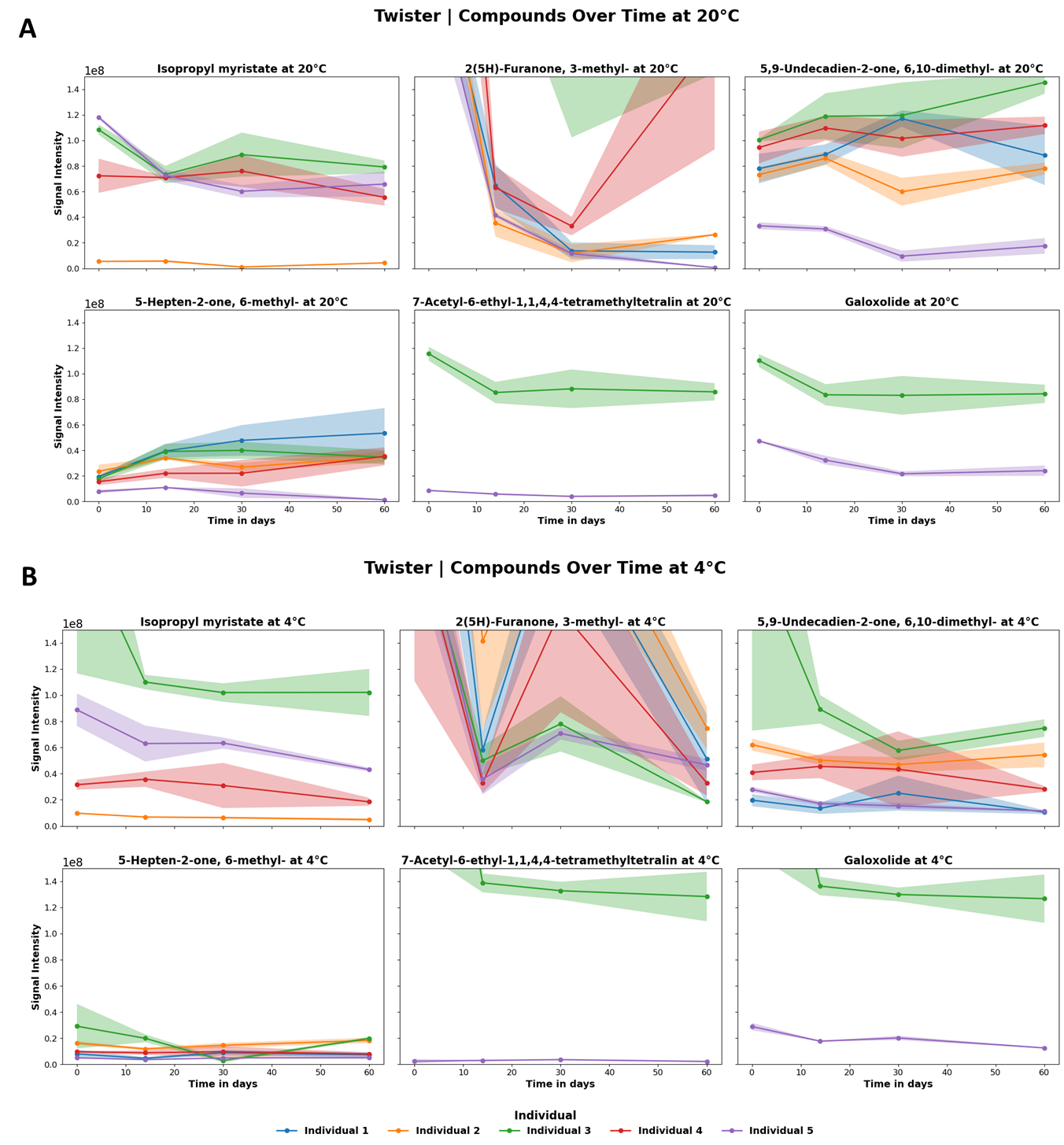


**Supplementary Figure 3 | Study 4:** VOC behavior over a 60-day period for different compounds using Twister as the capturing material (a) at 20°C and (b) at 4°C. Each graph shows the temporal variation in VOC signal intensity for individual compounds, with separate lines representing different compounds. The mean values are depicted by solid lines, and the standard error of the mean (SEM) is shown as shaded areas around each line. Different individuals are color-coded, and the legend corresponds to each individual.
